# Decreased Structural Connectivity Between Thalamic Nuclei and Hippocampus in Temporal Lobe Epilepsy—A Diffusion Tensor Imaging‐Based Study

**DOI:** 10.1111/ene.70040

**Published:** 2025-01-11

**Authors:** Mehmet S. Yildirim, Radheshyam Stepponat, Florian Ph. S. Fischmeister, Matthias Tomschik, Victor Schmidbauer, Farjad Khalaveh, Johannes Koren, Christoph Baumgartner, Ekaterina Pataraia, Silvia Bonelli, Karl Rössler, Gregor Kasprian, Christian Dorfer

**Affiliations:** ^1^ Department of Neurosurgery Medical University of Vienna Vienna Austria; ^2^ Developmental and Interventional Neuroimaging Lab (DINLAB), Department of Biomedical Imaging and Image‐Guided Therapy Medical University of Vienna Vienna Austria; ^3^ Division of Neuroradiology and Musculoskeletal Radiology Medical University of Vienna Vienna Austria; ^4^ Department of Neurology Clinic Hietzing Vienna Austria; ^5^ Karl Landsteiner Institute for Clinical Epilepsy Research and Cognitive Neurology Vienna Austria; ^6^ Department of Neurology Medical University of Vienna Vienna Austria

**Keywords:** anterior thalamic nuclei, diffusion tensor imaging, hippocampal sclerosis, structural brain network, temporal lobe epilepsy

## Abstract

**Background:**

Temporal lobe epilepsy (TLE) can lead to structural brain abnormalities, with thalamus atrophy being the most common extratemporal alteration. This study used probabilistic tractography to investigate the structural connectivity between individual thalamic nuclei and the hippocampus in TLE.

**Methods:**

Thirty‐six TLE patients who underwent pre‐surgical 3 Tesla magnetic resonance imaging (MRI) and 18 healthy controls were enrolled in this study. Patients were subdivided into TLE with HS (TLE‐HS) and MRI‐negative TLE (TLE‐MRneg). Tractography and whole brain segmentation, including thalamus parcellation, were performed to determine the number of streamlines per mm^3^ between the thalamic nuclei and hippocampus. Connectivity strength and volume of regions were correlated with clinical data.

**Results:**

The volume of the entire thalamus ipsilateral to seizure onset was significantly decreased in TLE‐HS compared to controls (Mann–Whitney‐*U* test: *p*_FDR_ < 0.01) with the anterior thalamic nuclei (ANT) as important contributor. Furthermore, decreased ipsilateral connectivity strength between the hippocampus and ANT was detected in TLE‐HS (*p*_FDR_ < 0.01) compared to TLE‐MRneg and controls which correlated negatively with the duration of epilepsy (*ρ* = −0.512, *p* = 0.025) and positively with seizure frequency (*ρ* = 0.603, *p* = 0.006). Moreover, ANT volume correlated negatively with epilepsy duration in TLE‐HS (*ρ* = −0.471, *p* = 0.042).

**Conclusions:**

ANT showed atrophy and decreased connectivity in TLE‐HS, which correlated with epilepsy duration and seizure frequency. Understanding the dynamics of epileptogenic networks has the potential to shed light on surgery‐resistant epilepsy and refine the selection process for ideal neurosurgical candidates, consequently enhancing post‐surgical outcomes.

**Abbreviations**
ANT: anterior thalamic nuclei
CSD: constrained spherical deconvolution
DBS: deep brain stimulation
DRE: drug‐resistant epilepsy
DTI: diffusion tensor imaging
HS: hippocampal sclerosis
INT: intralaminar thalamic nuclei
LNT: lateral thalamic nuclei
MNT: medial thalamic nuclei
MRI: magnetic resonance imaging
PNT: posterior thalamic nuclei
SEEG: stereoelectroencephalography
TE: time to echo
TLE: temporal lobe epilepsy
TLE‐HS: temporal lobe epilepsy with hippocampal sclerosis
TLE‐MRneg: MRI‐negative temporal lobe epilepsy
TR: repetition time
VEEG: video‐electroencephalogram
VNT: ventral thalamic nuclei

## 1 Introduction

Temporal lobe epilepsy (TLE) represents the most common form of epilepsy [1], and up to 40% of the patients do not respond to anti‐seizure medication [2]. While hippocampal sclerosis (HS) is the most common morphological substrate associated with medically‐refractory mesial TLE [3], extratemporal structural abnormalities were preferentially observed in the thalamus [4]. Temporal lobe resections in various forms have been proven effective revealing long‐term seizure freedom rates of about 70% [5]. In addition, other invasive modalities, including deep brain stimulation (DBS) [6, 7], responsive neurostimulation [8], and vagus nerve stimulation [9] have been introduced as alternatives for specific patient populations. The selection of any of these various treatment forms is, however, influenced by many factors and is often complicated by a limited understanding of the epilepsy network and the critical hubs involved. Thus, remaining or recurring seizures after invasive interventions still confront the treating physicians with challenges.

The anterior thalamic nuclei (ANT), the centromedian nucleus of the thalamus, and the hippocampus are often used for neuromodulation in drug‐resistant epilepsy (DRE) [6, 10, 11, 12]. While a median reduction in seizure frequency of up to 75% is reported [6, 10, 13], ideal patient and target region selections remain challenging due to the lack of in‐depth understanding of the underlying epileptogenic network [11].

In seeking to understand the substrates underlying these networks in TLE, functional and structural alterations in the temporal lobe and extratemporal areas, including both cortical and subcortical regions, have been investigated [4, 12]. There is compelling evidence that functional thalamus connectivity represents a contributing factor to the post‐surgical failure to achieve seizure freedom [14]. While a gross volume reduction of the ipsilateral and contralateral thalamus was encountered in 61% and 50% of TLE patients respectively [4], studies elucidating the structural hippocampal‐thalamic pathway in TLE are scarce [15, 16, 17].

Diffusion tensor imaging (DTI)‐based tractography allows for a non‐invasive in vivo investigation of white matter pathways. Studies using DTI in TLE revealed partially conflicting results regarding the connectivity between thalamus and temporal lobe structures [15, 16, 17]. In these studies, network investigation was conducted either for the entire thalamus or the cortico‐thalamic connected segment. To the best of our knowledge, no recent study has investigated the structural connectivity between the hippocampus and individual thalamic nuclei in TLE. A better understanding of the phenotype of the epileptic disorders and the underlying pathological networks and brain structures involved, however, is important for improving the selection process of candidates and types of surgical therapy, in order to open new avenues in the treatment of DRE.

Therefore, this study aimed to investigate the structural connectivity differences of the individual thalamic nuclei in patients with TLE and ipsilateral HS and TLE patients without any magnetic resonance imaging (MRI) alterations. For this purpose, probabilistic tractography and whole brain segmentation, including thalamus parcellation, were created and compared to healthy controls.

## 2 Materials and Methods

### 2.1 Ethical Approval

The local ethics committee (Medical University of Vienna, Vienna, Austria) approved the protocol of this retrospective study, which was conducted following the Helsinki Declaration of 1975 (EC‐Nr.: 1141/2023; 1955/2020).

### 2.2 Study Cohort

Between September 2019 and December 2023, pre‐surgical 3 Tesla MRI was performed in 87 patients with TLE at the Division of Neuroradiology in a tertiary care hospital. The imaging was conducted in accordance with the recommendations of the International League Against Epilepsy (ILAE) [18]. Epilepsy classification and localization of the seizure onset zone was determined based on seizure semiology, video‐electroencephalogram (VEEG), and MRI alterations in according to the ILAE classification scheme [19]. The imaging assessments were performed by a neuroimaging expert with 20 years of experience (G.K.).

**FIGURE 1:**
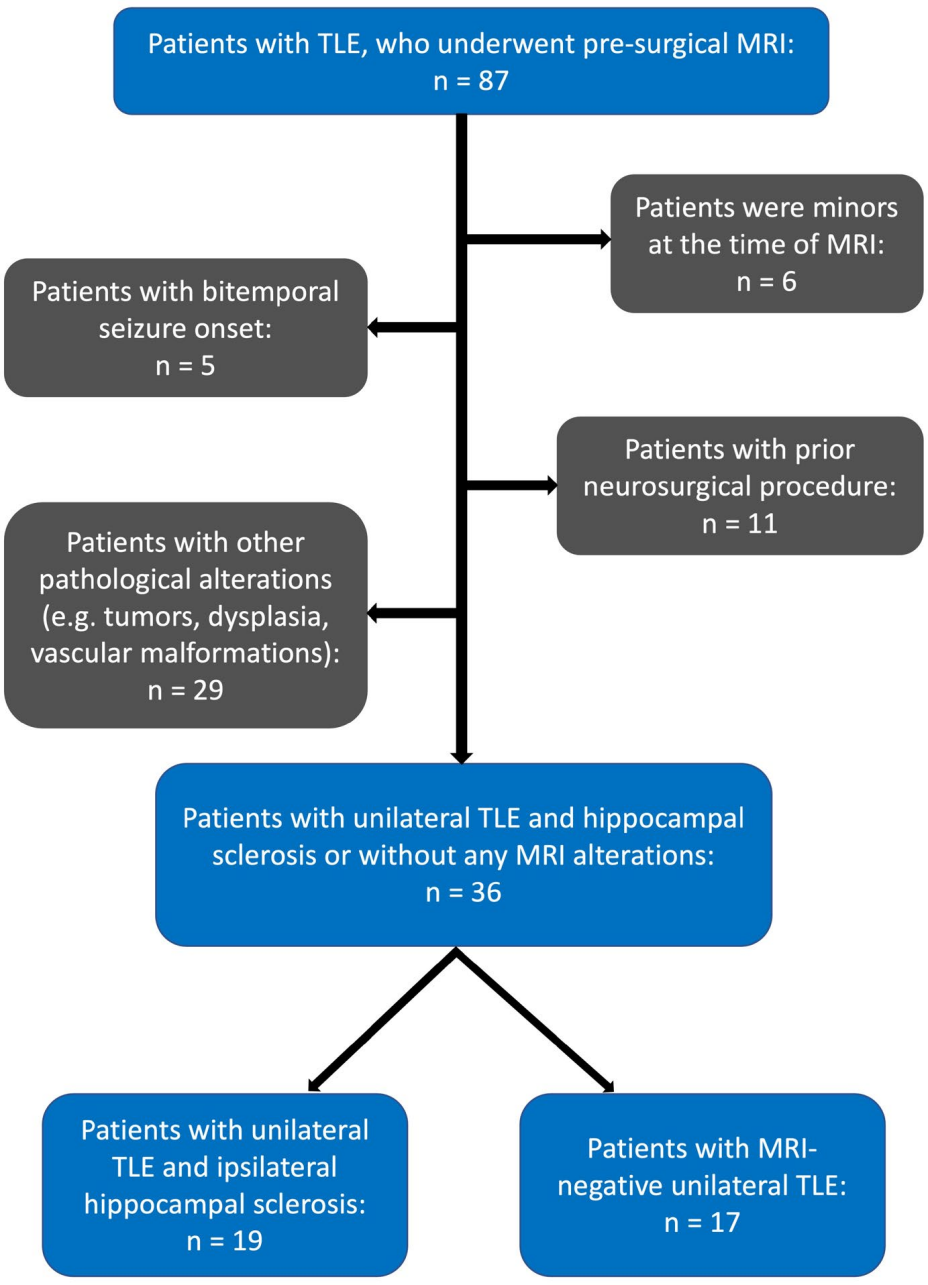
The selection of patient samples is shown by the flow diagram. 87 patients with temporal lobe epilepsy (TLE) underwent pre‐surgical 3 Tesla magnetic resonance imaging (MRI) between September 2019 and December 2023. Patients who were minors during the pre‐surgical MRI examination were excluded from this study (6/87). Moreover, subjects who had previously undergone neurosurgery (11/87) or bitemporal seizure onset (5/87) were also excluded. Furthermore, patients with other or additional pathological alterations detected alongside hippocampal sclerosis by MRI were excluded. Finally, a total of 36 patients were included in this study. Patients were further divided into two groups: (1) Unilateral TLE with ipsilateral hippocampal sclerosis; and (2) MRI‐negative TLE.

Only patients with unilateral HS as the sole visible alteration or with no visible pathologies detectable on MRI were included in this study. Patients with other pathologies alongside HS (e.g., tumors, vascular malformatios, dysplasia or temporal lobe white matter blurring) were excluded (Figure 1). Patients were divided into two groups: (1) unilateral TLE with ipsilateral HS (TLE‐HS); and (2) MRI‐negative unilateral TLE (TLE‐MRneg). In the TLE‐MRneg cohort, 13 patients were identified with seizure onset localized to the mesial temporal lobe based on findings from VEEG, positron emission tomography (PET) scans, and stereoelectroencephalography (SEEG). Neocortical TLE was identified in one patient. For three patients, differentiation between mesial and neocortical TLE remained inconclusive. T1‐weighted and DTI data of 18 healthy controls were acquired using the same MRI system. Table 1 gives an overview of the demographic and clinical data of the included subjects.

**TABLE 1:** Demographic and clinical data of included participants.

Characteristics	TLE‐HS (*n* = 19)	TLE‐MRneg (*n* = 17)	Healthy controls (*n* = 18)	*p* ^a^
Sex				
Female	9	8	7	0.845
Male	10	9	11	
Mean age (years) at MRI	42 ± 12	33 ± 9	30 ± 7	0.002
Seizure duration in years (median)	15	9		0.053
Number of focal seizures per year (median)	14	54^b^		0.119
Seizure onset zone				
Left temporal	12	4		
Right temporal	7	13		
Histological data				
HS Type I^c^	9	0		
HS Type II^c^	5	0		
FCD Type IIa^d^	0	1		
Gliosis and epileptogenic alterations^e^	12	4		
No epilepsy surgery performed	5	12		

Abbreviations: FCD, focal cortical dysplasia; HS, hippocampal sclerosis; TLE‐HS, temporal lobe epilepsy with hippocampal sclerosis; TLE‐MRneg, MRI‐negative temporal lobe epilepsy.

^a^ Data were compared using the Pearson χ ^2^, the Mann–Whitney‐*U*, or One‐way ANOVA for group homogeneity.

^b^ The seizure frequency in one patient with TLE‐MRneg could not be determined (*n* = 16).

^c^ According to International League against Epilepsy (ILAE) classification (2013) [20].

^d^ According to International League against Epilepsy (ILAE) classification (2011) [21].

^e^ In case of hippocampal sclerosis, gliosis and epileptogenic alterations were additionally detected in12 histological preparations.

### 2.3 MRI Data Acquisition

MR imaging data acquisition of all patients and healthy controls was performed using an identical MRI protocol on a 3 Tesla Philips Achieva scanner with a 32‐channel head coil. High‐resolution T1‐weighted sequences [Repetition time (TR)/Time to echo (TE) 8.1/3.7 ms; flip angle: 8°; slice thickness: 1 mm; voxel size: 1 × 0.75 × 0.75 mm; matrix: 320 × 320 × 180] were acquired as anatomical imaging data. A multi‐shell diffusion‐weighted MRI acquisition [TR/TE 3150/89 ms; 64 gradient‐encoded directions; *b*‐values: 0, 1000, 2000 s/mm^2^; flip angle: 90°; voxel size: 1.56 × 1.56 × 2 mm; matrix: 144 × 144 × 66] was performed for probabilistic tractography.

### 2.4 Probabilistic Tractography and Structural Connectivity Analysis

A probabilistic tractography was performed by creating 50 million streamlines using the MRtrix3 software package (MRtrix3; Version 3.0.4) [22]. The “spherical‐deconvolution informed filtering of tractograms” (SIFT) method was performed for optimization of the reconstructed streamlines by matching the tractography streamlines with the fiber densities using the constrained spherical deconvolution (CSD) model [23]. Preprocessing of the diffusion‐weighted data was done using the default procedure of the FSL eddy tool for multi‐shell DTI (FMRIB Software Library, Analysis Group, FMRIB, Oxford, UK; Version 6) [24]. Co‐registration of the diffusion‐weighted imaging and anatomical data as the reference image was performed using linear transformation. Whole brain segmentation of the T1‐weighted acquisitions and volumetric determination was performed using the Freesurfer neuroimaging software package (https://surfer.nmr.mgh.harvard.edu; Version 7.4.0). Cortical and subcortical parcellation was done based on the standard Freesurfer atlas [25, 26, 27, 28]. Freesurfer software was also used for the segmentation of thalamic nuclei into 25 parcellations in each hemisphere based on ex vivo MR imaging of brains and histological data [29]. All thalamic parcellations were merged into a total of six different regions of nuclei: ANT, including anterior medial, anterior dorsal, and anterior ventral nuclei; intralaminar thalamic nuclei (INT); lateral thalamic nuclei (LNT); medial thalamic nuclei (MNT); posterior thalamic nuclei (PNT); ventral thalamic nuclei (VNT) [29]. The structural connectivity network was created in individual patient's brain based on tractography streamlines and brain segmentation.

**FIGURE 2:**
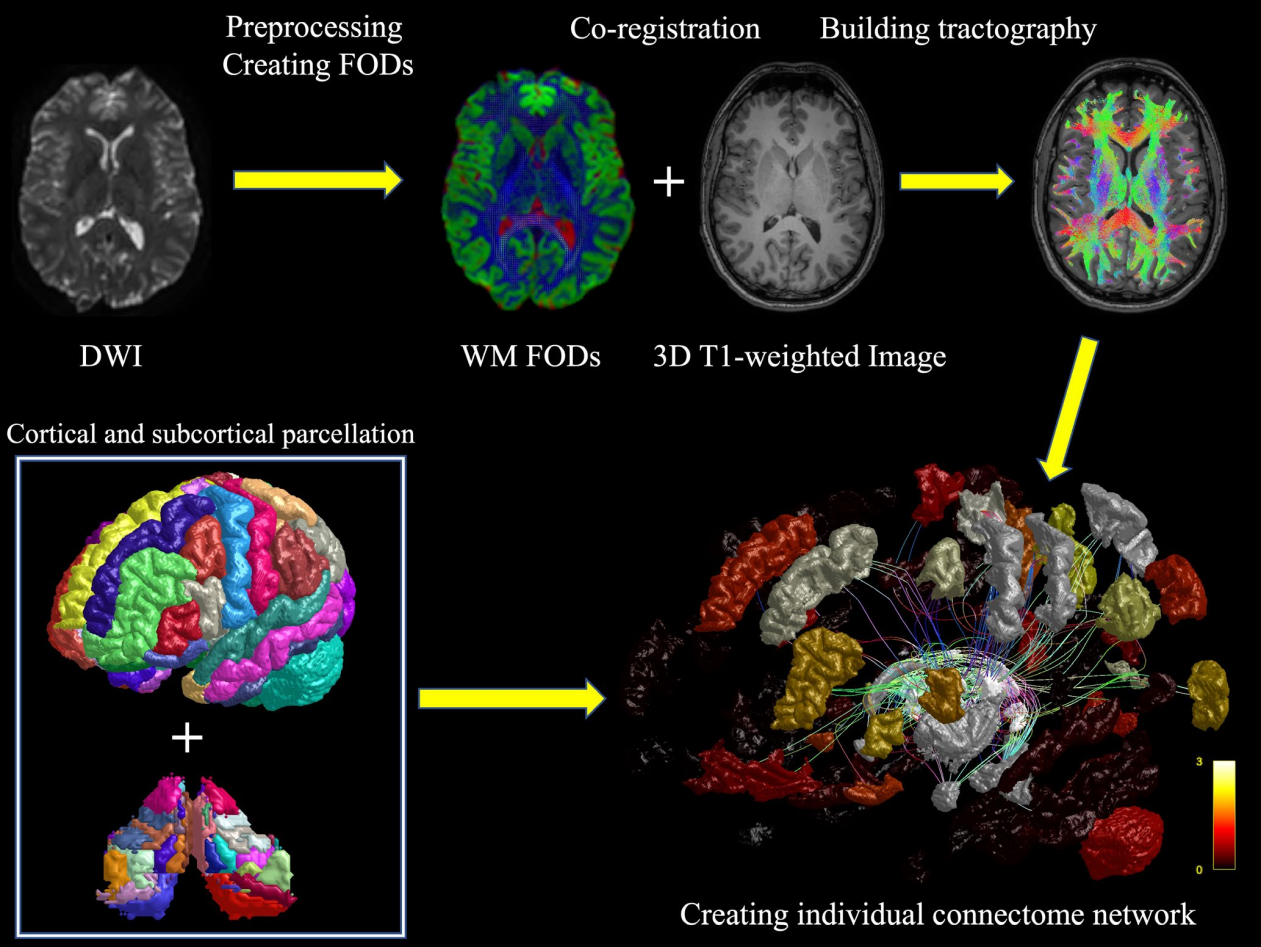
Connectivity analysis was performed using the MRtrix3 software package, FSL, and Freesurfer for cortical and subcortical brain segmentation (see image in the bottom left). First, preprocessing of the diffusion‐weighted imaging (DWI) data was performed, followed by the creation of fiber orientation distributions (FODs). Afterward, co‐registration of the diffusion‐weighted imaging and anatomical T1‐weighted imaging data was performed. The tractography was done by creating 50 million streamlines (see images in the first line). Finally, the connectivity network was built in individual patient's brain based on anatomical segmentation and tractography. The image in the bottom right shows the visualization of the connectivity network of the left‐hemispheric thalamus. Edges (streamlines) are colored by direction (red: Left‐to‐right, commissural fibers; green: Anterior‐to‐posterior, association fibers; blue: Cranial‐to‐caudal, projection fibers). Nodes (brain regions) are colored according to their connectivity strength with the seed point. The colored bar indicates the number of streamlines per volume connecting the seed point with the target region.

The hippocampus was defined as the seed point, and the entire thalamus, including individual thalamic nuclei, were set as target regions. The number of streamlines connecting two brain regions was divided by the total volume of the seed and target region for volume‐corrected comparison between groups. The connectivity strength is expressed in streamlines per mm^3^. A simplified version of the network analysis steps is presented in Figure 2.

### 2.5 Statistical Analyses

The statistical analyses were performed using SPSS Statistics for Mac (Version 29.0; IBM Armonk, NY, USA). Graphical methods, including Q‐Q plots and histograms, along with analytical tests such as the Shapiro–Wilk test, were used to assess the normality of the entire data.

Data of patients were divided into ipsilaterally and contralaterally to the seizure onset hemisphere and were compared separately with each hemisphere of controls due to differences between the data from the left and right hemispheres in the control group. The comparison of connectivity and volume of regions was performed using a 2 × 3 rank‐based non‐parametric analysis of variance (Kruskal–Wallis test) to determine significant effect differences between groups (first: patient's data was compared with the left hemisphere of controls; second: patient's data was compared with the right hemisphere of controls). Due to multiple comparisons, an adjusted *p*‐value of *p*_FDR_ < 0.05 (False discovery rate corrected *p*‐value: *p*_FDR_) was considered statistically significant. Mann–Whitney‐*U* test post hoc analysis was conducted to test specific hypotheses. Table 2 gives an overview of the results from the Kruskal–Wallis test.

**TABLE 2:** Results from the Kruskal–Wallis test.

Brain structure^a^	TLE‐HS		TLE‐MRneg		Healthy controls		Kruskal–Wallis test: *T*; *p*_FDR_‐value^b^	Kruskal–Wallis test: *T*; *p*_FDR_‐value^c^
	Mean	Median	Mean	Median	Mean	Median		
Volume: Entire thalamus ipsilateral^d^	6291 ± 907	6314	6959 ± 599	7000	7496 ± 609^e^	7449^e^	18.2; 0.002	14.8; 0.004
Volume: Entire thalamus contralateral^d^	6722 ± 640	6651	7061 ± 688	7184	7284 ± 470^f^	7290^f^	10.5; 0.015	7.2; 0.054
Volume: ANT ipsilateral^d^	109 ± 27	106	122 ± 15	121	133 ± 16^e^	125^e^	11.6; 0.010	16.5; 0.003
Volume: ANT contralateral^d^	119 ± 19	118	122 ± 15	117	141 ± 16^f^	141^f^	6.9; 0.057	14.1; 0.005
Volume: MNT ipsilateral^d^	983 ± 127	1003	1040 ± 77	1035	1139 ± 97^e^	1159^e^	14.6; 0.004	14.4; 0.004
Volume: MNT contralateral^d^	1035 ± 124	1061	1047 ± 93	1030	1117 ± 67^f^	1135^f^	8.6; 0.032	6.1; 0.071
Volume: PNT ipsilateral^d^	2034 ± 333	2032	2281 ± 212	2232	2528 ± 239^e^	2517^e^	22.1; < 0.001	8.6; 0.032
Volume: PNT contralateral^d^	2190 ± 231	2201	2389 ± 292	2397	2334 ± 210^f^	2334^f^	12.2; 0.008	4.5; 0.141
Volume: VNT ipsilateral^d^	2656 ± 397	2683	2953 ± 352	2930	3089 ± 291^e^	3030^e^	10.9; 0.013	12.6; 0.007
Volume: VNT contralateral^d^	2822 ± 277	2775	2933 ± 348	2925	3092 ± 244^f^	3065^f^	6.2: 0.070	6.2; 0.070
Volume: LNT ipsilateral^d^	122 ± 33	115	127 ± 20	124	151 ± 19^e^	149^e^	12.3; 0.008	7.3; 0.053
Volume: LNT contralateral^d^	135 ± 30	125	136 ± 20	140	147 ± 27^f^	148^f^	6.9; 0.057	3.3; 0.227
Volume: INT ipsilateral^d^	388 ± 62	385	435 ± 45	434	458 ± 43^e^	450^e^	13.4; 0.006	12.1; 0.008
Volume: INT contralateral^d^	421 ± 52	399	435 ± 55	422	453 ± 41^f^	454^f^	6.9; 0.057	5.9; 0.074
Volume: HC ipsilateral^d^	2619 ± 378	2565	3604 ± 313	3566	3698 ± 308^e^	3606^e^	35.6; < 0.001	35.9; < 0.001
Volume: HC contralateral^d^	3317 ± 484	3376	3512 ± 301	3488	3689 ± 244^f^	3625^f^	6.8; 0.057	7.9; 0.045
Connectivity: HC‐entire thalamus ipsilateral^g^	15.9 ± 4.5	15.2	16.1 ± 3.3	16.3	17.4 ± 3.9^e^	16.9^e^	1.9; 0.422	2.2; 0.384
Connectivity: HC‐entire thalamus contralateral^g^	16.2 ± 4.4	17.2	16.2 ± 3.5	16.6	17.5 ± 3.1^f^	17.1^f^	1.0; 0.635	1.3; 0.555
Connectivity: HC‐ANT ipsilateral^g^	0.3 ± 0.4	0.2	1.1 ± 1.0	0.8	0.8 ± 0.6^e^	0.6^e^	15.0; 0.004	15.8; 0.004
Connectivity: HC‐ANT contralateral^g^	0.6 ± 0.6	0.2	0.8 ± 0.8	0.5	0.8 ± 0.5^f^	0.6^f^	4.4; 0.141	7.0; 0.057
Connectivity: HC‐MNT ipsilateral^g^	1.2 ± 1.0	0.9	1.5 ± 0.8	1.4	2.2 ± 1.3^e^	1.8^e^	8.6; 0.032	6.1; 0.070
Connectivity: HC‐MNT contralateral^g^	1.2 ± 0.8	1.1	1.6 ± 0.9	1.2	1.9 ± 1.2^f^	1.5^f^	7.6; 0.048	4.2; 0.154
Connectivity: HC‐PNT ipsilateral^g^	28.6 ± 8.8	27.6	25.9 ± 5.5	24.5	26.1 ± 5.9^e^	24.0^e^	2.3; 0.357	1.5; 0.508
Connectivity: HC‐PNT contralateral^g^	26.6 ± 7.9	27.0	26.3 ± 6.1	26.5	27.3 ± 4.8^f^	27.0^f^	0.2; 0.906	0.4; 0.840
Connectivity: HC‐VNT ipsilateral^g^	3.0 ± 1.2	2.7	2.3 ± 1.4	2.0	3.9 ± 1.6^e^	3.4^e^	13.6; 0.005	6.3; 0.070
Connectivity: HC‐VNT contralateral^g^	2.3 ± 1.2	2.2	2.7 ± 0.9	2.4	2.8 ± 0.8^f^	2.7^f^	13.1; 0.006	2.8; 0.292
Connectivity: HC‐LNT ipsilateral^g^	0.2 ± 0.3	0.1	0.3 ± 0.3	0.2	0.4 ± 0.3^e^	0.4^e^	8.6; 0.032	5.0; 0.113
Connectivity: HC‐LNT contralateral^g^	0.2 ± 0.3	0.1	0.3 ± 0.4	0.2	0.4 ± 0.3^f^	0.2^f^	7.8; 0.045	3.8; 0.185
Connectivity: HC‐INT ipsilateral^g^	0.5 ± 0.4	0.4	0.3 ± 0.3	0.3	0.6 ± 0.4^e^	0.6^e^	6.2; 0.070	3.7; 0.190
Connectivity: HC‐INT contralateral^g^	0.4 ± 0.4	0.3	0.4 ± 0.3	0.3	0.5 ± 0.3^f^	0.4^f^	5.3; 0.100	2.0; 0.414

Abbreviations: ANT, anterior thalamic nuclei; HC, hippocampus; INT, intralaminar thalamic nuclei; LNT, lateral thalamic nuclei; MNT, medial thalamic nuclei; PNT, posterior thalamic nuclei; TLE‐HS, temporal lobe epilepsy with hippocampal sclerosis; TLE‐MRneg, MRI‐negative temporal lobe epilepsy; VNT, ventral thalamic nuclei.

^a^ Brain regions/pathways were classified as either ipsilateral or contralateral to the seizure focus in patients. For controls, data is separated based on anatomical location (left and right hemisphere).

^b^ The patients' data were compared with the results from the left hemisphere of controls.

^c^ The patients' data were compared with the results from the right hemisphere of controls.

^d^ Volume in mm^3^.

^e^ Results of the left hemisphere in case of controls.

^f^ Results of the right hemisphere in case of controls.

^g^ Number of streamlines per mm^3^ combined volume of the seed and target region.

Hippocampal‐thalamic connectivity strength and volume of regions of interest were correlated with epilepsy duration and number of focal seizures per year using Spearman's rank correlation test.

## 3 Results

### 3.1 Participant Characteristics

From an initial cohort of 87 patients, 51 patients were excluded from this study to meet the stringent inclusion criteria. A total of 36 patients with unilateral TLE (TLE‐HS: 19; TLE‐MRneg: 17) were included in this study (Figure 1). Epilepsy surgery was performed in 19 of 36 patients. Histological data are given in Table 1. Eighteen healthy subjects were enrolled as a control group.

No differences between groups were found in terms of sex distribution, seizure duration, and seizure frequency. Differences between groups were only found with respect to age. For details, please refer to Table 1.

Due to age‐related differences, a Quade non‐parametric analysis of covariance (ANCOVA) was conducted. The results are shown in Table S2. As the age‐corrected *p*‐values are very similar to the FDR‐corrected *p*‐values from the Mann–Whitney‐*U* test, only the results from the post hoc analysis will be presented and discussed in the following sections.

### 3.2 Volumes of the Thalamic Nuclei and Hippocampus

**FIGURE 3:**
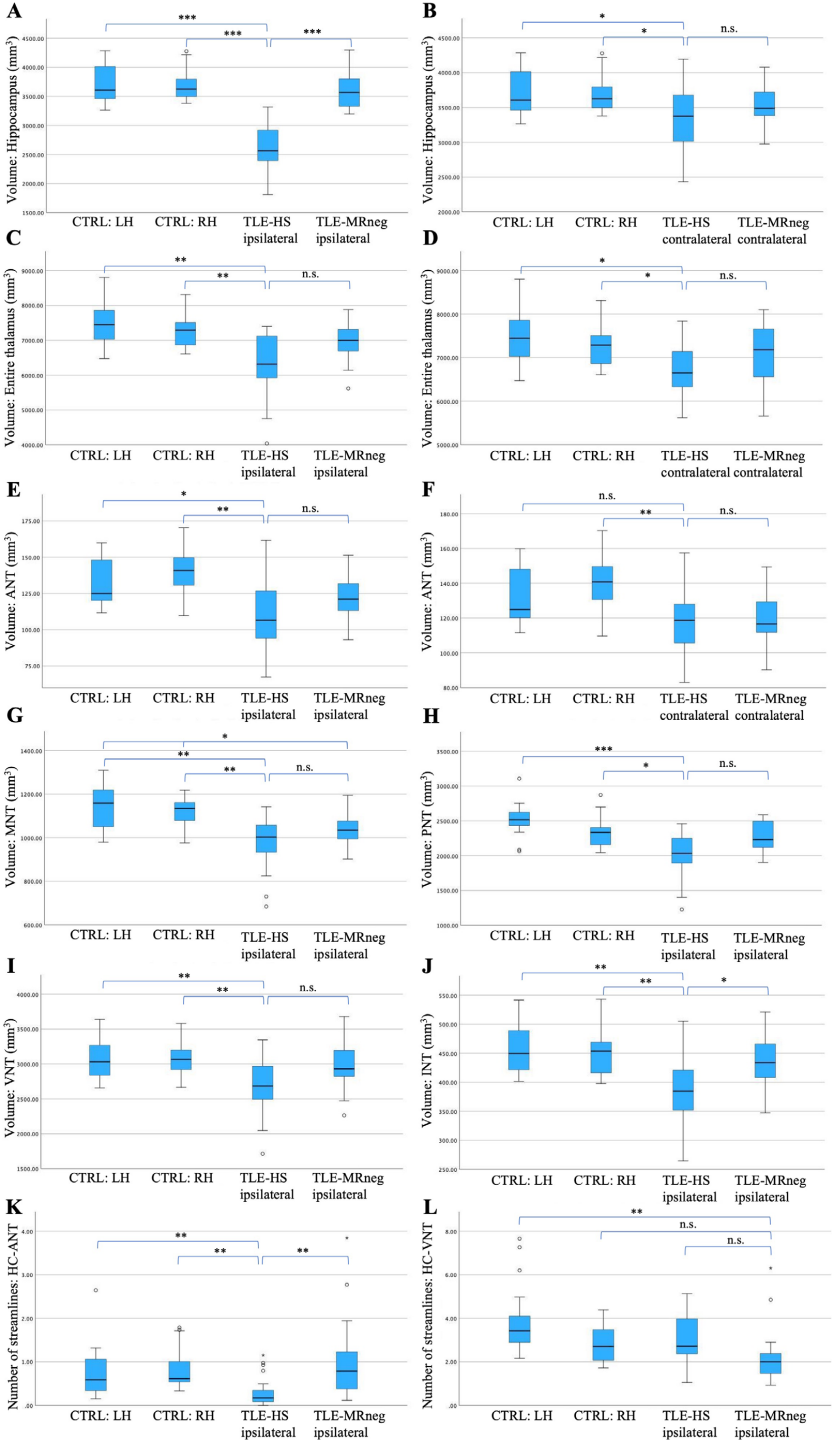
The boxplots show differences in ipsilateral (A) and contralateral (B) hippocampal volume; ipsilateral (C) and contralateral (D) volume of entire thalamus; ipsilateral (E) and contralateral (F) volume of anterior thalamic nuclei (ANT); ipsilateral volume of medial thalamic nuclei (MNT) (G), posterior thalamic nuclei (PNT) (H), ventral thalamic nuclei (VNT) (I) and intralaminar thalamic nuclei (INT) (J); and the number of streamlines between ipsilateral hippocampus and ANT (K) and VNT (L) between patients with temporal lobe epilepsy and hippocampal sclerosis (TLE‐HS), TLE without MRI alterations (TLE‐MRneg) and healthy controls (CTRL). Results of controls were divided into left‐ and right hemispheres (LH/RH). The black lines indicate the mean values. The most significant volume loss of thalamic nuclei was found in TLE‐HS, with greater differences observed in ANT, MNT, and INT compared to controls. *p_FDR_ < 0.05; **p_FDR_ < 0.01; ***p_FDR_ < 0.001; n.s., no significance.

The hippocampal volume ipsilateral to seizure onset was highly significantly smaller in TLE‐HS compared to TLE‐MRneg (Mann–Whitney‐*U* test, *p*_FDR_ < 0.001) and healthy controls (compared to both hemispheres: *p*_FDR_ < 0.001; see Figure 3A). These results are in accordance with the radiological diagnosis of HS. Moreover, a contralateral hippocampus atrophy in TLE‐HS was also observed compared to controls (left hemisphere: *p*_FDR_ = 0.049; right hemisphere: *p*_FDR_ = 0.038; see Figure 3B). The volume of the entire thalamus ipsilateral to epileptogenic focus was reduced in TLE‐HS compared to healthy controls (both hemispheres: *p*_FDR_ < 0.01; see Figure 3C), with a trend atrophy compared to TLE‐MRneg (*p*_FDR_ = 0.054; see Figure 3C). The contralateral thalamic volume also showed a decrease in TLE‐HS compared to controls (left hemisphere: *p*_FDR_ = 0.013; right hemisphere: *p*_FDR_ = 0.032; see Figure 3D). Furthermore, there was bilateral atrophy of ANT (ipsilateral compared to left hemisphere: *p*_FDR_ = 0.013, right hemisphere: *p*_FDR_ = 0.006; contralateral compared to left hemisphere: *p*_FDR_ = 0.054, right hemisphere: *p*_FDR_ = 0.008; see Figure 3E,F) and ipsilateral volume loss of MNT (both hemispheres: *p*_FDR_ < 0.01; see Figure 3G) observed in TLE‐HS compared to controls. A reduced volume of ipsilateral PNT (see Figure 3H), VNT (see Figure 3I), and INT (see Figure 3J) was also detected in TLE‐HS compared to controls.

In TLE‐MRneg, there was only a significant volume loss of the MNT ipsilateral to epileptogenic focus compared to controls (left hemisphere: *p*_FDR_ = 0.022; right hemisphere: *p*_FDR_ = 0.032; see Figure 3G). The results from the Mann–Whitney‐*U* test post hoc analysis are shown in Table S1.

### 3.3 Hippocampal‐Thalamic Structural Connectivity Differences

The structural connectivity analysis revealed a significantly lower number of streamlines per volume between ANT and hippocampus ipsilateral to seizure onset zone in TLE‐HS compared to TLE‐MRneg (*p*_FDR_ = 0.008) and controls (left hemisphere: *p*_FDR_ = 0.008; right hemisphere: *p*_FDR_ = 0.006; see Figure 3K). Moreover, there was a significant reduction of the ipsilateral connectivity between VNT and hippocampus in TLE‐MRneg compared to the corresponding left‐hemispheric pathway in healthy controls (*p*_FDR_ = 0.005; see Figure 3L), with a trend of connectivity decrease compared to the right hemisphere (*p*_FDR_ = 0.080). No significant differences were detected regarding connectivity between the hippocampus and other thalamic nuclei (Tables 2 and Table S1).

### 3.4 Correlations With Clinical Data

**FIGURE 4:**
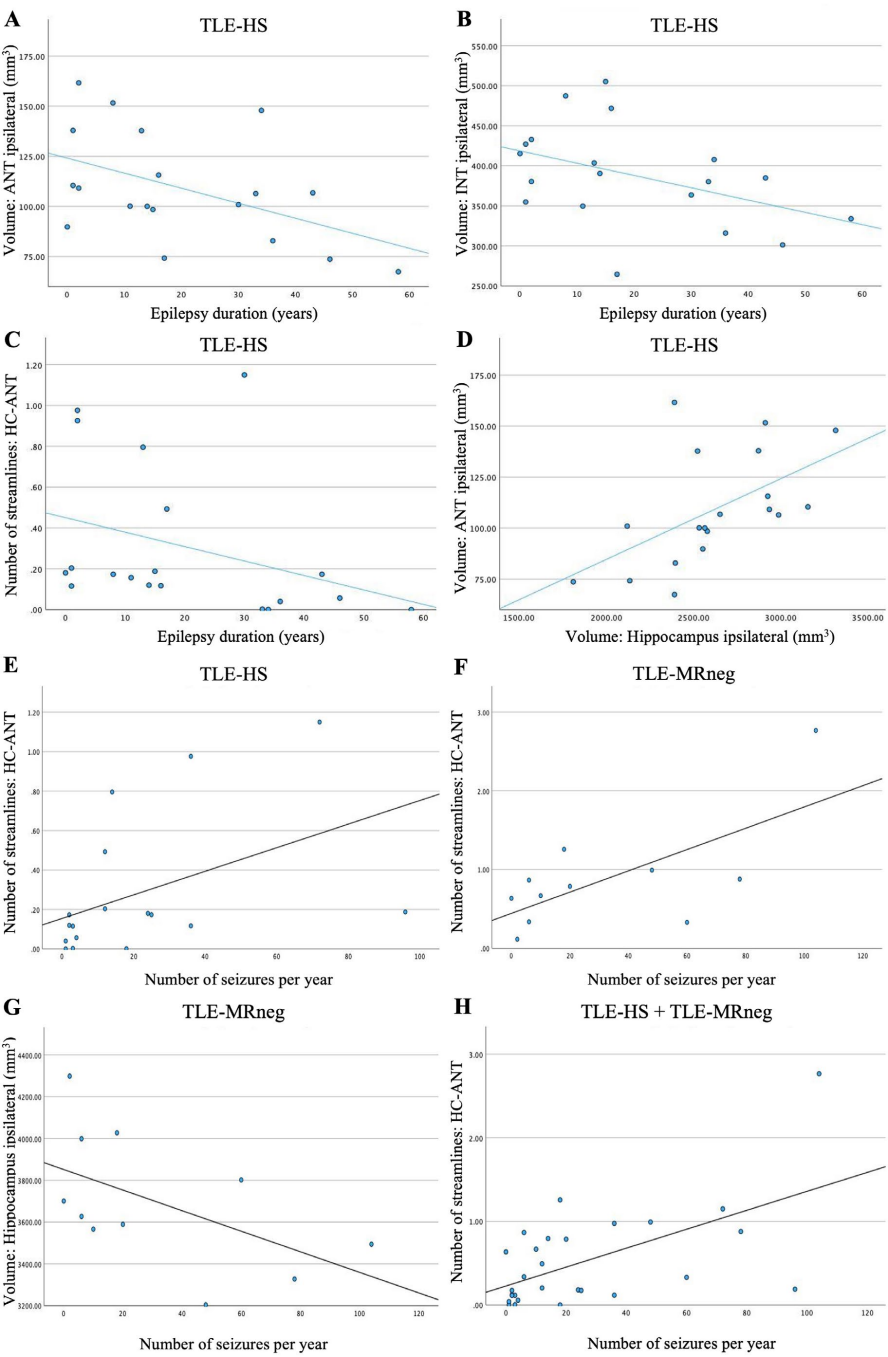
Clinical data was correlated with the volume of regions of interest and hippocampal‐thalamic connectivity. The epilepsy duration was negatively correlated with the ipsilateral volume of anterior thalamic nuclei (ANT) (A, Spearman's *ρ* = −0.471, *p* = 0.042), intralaminar thalamic nuclei (INT) (B, *ρ* = −0.467, *p* = 0.044), and number of streamlines between ipsilateral ANT and hippocampus (HC) (C, *ρ* = −0.512, *p* = 0.025) in patients with temporal lobe epilepsy and hippocampal sclerosis (TLE‐HS). Furthermore, the volume of the hippocampus ipsilateral to seizure onset was positively correlated with ANT volume (D, *ρ* = 0.535, *p* = 0.018). The number of focal seizures per year was positively correlated with ipsilateral connectivity between ANT and HC (E, *ρ* = 0.603, *p* = 0.006) in TLE‐HS. In patients with MRI‐negative temporal lobe epilepsy (TLE‐MRneg), no significant correlation between seizure frequency and ipsilateral ANT connectivity (F, *ρ* = 0.373, *p* = 0.154) could be detected. However, negative correlation between volume of ipsilateral HC and seizure frequency was found in TLE‐MRneg (G, *ρ* = −0.635, *p* = 0.008). Furthermore, seizure frequency was positively correlated with number of streamlines between HC and ANT ipsilateral to epileptogenic focus (H, *ρ* = 0.573, *p* < 0.001), when the two patient groups are combined (TLE‐HS + TLE‐MRneg).

The correlation analysis was limited to brain structures that showed statistical significance in the Kruskal–Wallis test (Table 2). There was a negative correlation between epilepsy duration and ANT volume (Spearman's *ρ* = −0.471, *p* = 0.042; see Figure 4A), INT volume (*ρ* = −0.467, *p* = 0.044; see Figure 4B), and connectivity strength of the hippocampal‐ANT pathway (*ρ* = −0.512, *p* = 0.025; see Figure 4C) ipsilateral to the seizure focus in patients with TLE‐HS. Moreover, hippocampal volume ipsilateral to the epileptogenic focus was correlated positively with the ipsilateral ANT (*ρ* = 0.535, *p* = 0.018; see Figure 4D) and PNT volume (*ρ* = 0.468, *p* = 0.043; Table 3) in TLE‐HS. In terms of seizure frequency, a positive correlation to ipsilateral connectivity between HC and ANT (*ρ* = 0.603, *p* = 0.006; see Figure 4E) was detected in TLE‐HS. In patients with TLE‐MRneg, there was no correlation between ANT connectivity and seizure frequency (*ρ* = 0.373, *p* = 0.154; see Figure 4F). However, a negative correlation between seizure frequency and ipsilateral hippocampal volume was found in TLE‐MRneg (*ρ* = −0.635, *p* = 0.008; see Figure 4G) (Table 3). Furthermore, the connectivity between ipsilateral HC and ANT correlated positively with seizure frequency in the combined patient group (TLE‐HS + TLE‐MRneg; *ρ* = 0.573, *p* < 0.001; see Figure 4H).

**TABLE 3:** Results from the correlation analysis.

Brain structures	TLE‐HS (CC/*p*‐value^a^)			TLE‐MRneg (CC/*p*‐value^a^)		
	SD^b^	SF^c^	HCV^d^	SD^b^	SF^c^	HCV^d^
Volume: Entire thalamus ipsilateral	−0.196/0.422	0.164/0.504	0.353/0.139	−0.216/0.404	0.012/0.965	−0.037/0.889
Volume: ANT ipsilateral	−0.471/0.042	0.235/0.333	0.535/0.018	−0.062/0.814	−0.230/0.391	0.444/0.074
Volume: MNT ipsilateral	−0.428/0.067	0.301/0.211	0.363/0.126	−0.108/0.681	0.097/0.720	−0.137/0.599
Volume: INT ipsilateral	−0.467/0.044	0.307/0.201	0.433/0.064	−0.207/0.426	0.058/0.832	0.208/0.422
Volume: PNT ipsilateral	−0.143/0.559	0.049/0.841	0.468/0.043	−0.156/0.550	0.035/0.896	−0.127/0.626
Volume: VNT ipsilateral	−0.145/0.554	0.178/0.465	0.365/0.124	−0.176/0.500	0.083/0.761	−0.054/0.837
HC‐ANT ipsilateral	−0.512/0.025	0.603/0.006	−0.295/0.221	0.317/0.216	0.373/0.154	−0.103/0.694
Volume: HC ipsilateral	−0.289/0.230	0.038/0.878	—	−0.125/0.633	−0.635/0.008	—

Abbreviations: ANT, anterior thalamic nuclei; CC, correlation coefficient; HC, hippocampus; HCV, hippocampal volume; INT, intralaminar thalamic nuclei; PNT, posterior thalamic nuclei; SD, seizure duration; SF, seizure frequency; TLE‐HS, temporal lobe epilepsy with hippocampal sclerosis; TLE‐MRneg, MRI‐negative temporal lobe epilepsy; VNT, ventral thalamic nuclei.

^a^ Correlation analysis was performed using Spearman's rank correlation.

^b^ Seizure duration in years since epilepsy diagnosis.

^c^ Seizure frequency in number of seizures per year.

^d^ Ipsilateral hippocampal volume.

## 4 Discussion

In this study, the structural connectivity between the hippocampus and individual thalamic nuclei was investigated in patients with TLE and ipsilateral HS or MRI‐negative TLE. The entire thalamus and nearly all thalamic subnuclei exhibited volume atrophy, particularly ipsilateral to the seizure onset zone in TLE‐HS compared to healthy controls. A significant reduction in ipsilateral structural connectivity between ANT and hippocampus was observed in TLE‐HS but not in TLE‐MRneg and controls. In contrast, connectivity loss of ipsilateral hippocampal‐VNT pathway was found in TLE‐MRneg but not in TLE‐HS. Moreover, the connectivity strength and volume of ANT were correlated negatively with the duration of epilepsy in TLE‐HS. There was also a positive correlation between seizure frequency and hippocampal‐ANT connectivity. These findings give insight into morphological substrates of TLE networks and their increasing maturation and damage to both distinct thalamic nuclei and hippocampal‐thalamic pathways.

Our findings are consistent with previous studies that reported bilateral thalamic volume loss [15, 16], including bilateral ANT atrophy in TLE [30]. In contrast to those studies, we investigated the thalamic alterations not only in comparison to healthy controls but also in comparison to patients with MRI‐negative TLE. Consequently, we were able to report that the thalamus is more severely affected in TLE‐HS, whereas TLE‐MRneg only showed atrophy of the MNT compared to controls. Prior research suggests an alteration in the structural pathways between the thalamus and mesial temporal lobe structures [15, 16, 17], without, however, taking into account the network involving individual thalamic nuclei. In the present study, we showed for the first time decreased structural connectivity between hippocampus and ANT, which correlated with epilepsy duration and seizure frequency, suggesting that the ANT serves as a hub within the epileptogenic network. Our results support the hypothesis of excitotoxic thalamic damage induced by seizure propagation through the hippocampal‐thalamic pathway [4, 16], especially in TLE patients with HS. In this context, the ANT appears to be a more vulnerable thalamic structure, exhibiting greater susceptibility to atrophy and connectivity loss.

The thalamus first emerged as a promising therapeutic target region in epilepsy treatment over 70 years ago based on animal studies in which a crucial role in seizure generalization was observed [12]. However, this hypothesis could not be confirmed in patients with TLE [31]. Recent clinical studies in humans have demonstrated a key role of the thalamus in epilepsy with focal seizure onset [6, 13, 32], regardless of secondary generalization. There is evidence that thalamic connectivity is associated with post‐surgical outcomes in TLE [14, 30]. Increased functional connectivity of the bilateral thalamus was observed in patients with seizure recurrence 1 year after anterior temporal lobectomy compared to healthy controls and seizure‐free patients [14].

The ANT has become the most important extratemporal target region for neuromodulation in DRE [6, 12] and is connected to the retrosplenial and subicular cortex and via the mammillothalamic tract with the mammillary body, which are part of the “Papez‐circuit” [12]. DBS of ANT can interrupt seizure propagation by desynchronizing this circuit [12]. Schaper et al. [33] examined 20 patients 1 year after ANT‐DBS and observed a significantly shorter distance of the electrodes to the ANT and mammillothalamic tract in patients with a larger reduction of seizure frequency. The most recent long‐term neurostimulation study revealed a median reduction of seizure frequency of 75% at least 7 years in epilepsy patients with DBS of bilateral ANT. Over 60% of the included patients suffered from drug‐resistant TLE with no significant differences in seizure reduction compared to seizure onset in the frontal or parietal lobe [13].

Keller et al. [15] investigated the thalamo‐cortical connectivity and detected a reduction in the mean number of streamlines of the bilateral thalamo‐temporal pathway, as well as bilateral volume loss in the connected thalamic segment in TLE. Decrease of thalamo‐frontal connectivity [34] and atrophy of connected volume was also observed in the thalamus segment connected to the hippocampus in TLE‐HS [16]. These findings are consistent with our data indicating bilateral thalamic volume loss and a decrease of connectivity between ANT and hippocampus in TLE‐HS. Conversely, another study indicated enhanced connectivity between the thalamus and ipsilateral hippocampus in TLE‐HS [17], a finding not confirmed by our results. The contrasting conclusion may be attributed to Dinkelacker et al.'s use of a different tractography model based on Bayesian probability theory and single‐shell DTI acquisitions, which provide less detailed information on white matter structures, including details on crossing fibers [35].

In more recent studies, atrophy of bilateral thalamic subnuclei could be shown in patients with DRE [36], including bilateral ANT detected on a 7T MR system [30]. In right‐hemispheric TLE with HS, a decreased volume of ipsilateral ANT and parafascicular nucleus was detected. However, patients with left‐sided TLE and HS showed bilateral atrophy of the parafascicular nucleus [36]. Bernhardt et al. [37] detected ipsilateral volume loss of the anterior, medial, and posterior division of the thalamus in TLE. In our study, we investigated thalamic subnuclei, including ANT, MNT, and INT that contains the parafascicular nucleus. Prior results are partially in line with our findings that indicate bilateral ANT atrophy as well as INT and MNT volume reduction ipsilateral to seizure onset in TLE‐HS. Sinjab et al. [38] analyzed postmortem histological data, identifying neuronal loss in ventrolateral and mediodorsal thalamic nucleus in TLE‐HS. Our data further support these results, indicating atrophy in the VNT (including ventrolateral nucleus) and MNT (including mediodorsal nucleus).

Terry et al. [39] used a computer simulation to investigate EEG data and found that decreasing connectivity between brain regions led to an increased frequency of epileptogenic activity. This aligns with numerous studies that have detected widespread structural and functional connectivity loss in DRE [40, 41], including reduced connectivity between the thalamus and temporal lobe regions [15, 16]. Although decreased ANT connectivity in TLE‐HS was shown in this study, it correlated positively with the number of focal seizures. Our results are thus in line with a recent study by Giampiccolo et al. [42], suggesting that thalamic network disconnection may lead to seizure freedom. These findings are further supported by an SEEG study that detected decreased functional connectivity within the epileptogenic network during high‐frequency stimulation in patients with DRE undergoing ANT‐DBS [43]. Furthermore, we observed signficantly higher ANT‐hippocampal connectivity in TLE‐MRneg compared to TLE‐HS, which also exhibited a trend of higher seizure frequency (median number of focal seizure per year, TLE‐MRneg vs. TLE‐HS: 54 vs. 14, Table 1), indicating that a more intact thalamic network is associated with a more severe form of the disease. This corroborates with recent evidence, suggesting that better post‐surgical outcomes to be associated with non‐lesional epilepsy after DBS [44, 45, 46]. Piacentino et al. [44], for instance, investigated patients at least 3 years after ANT‐DBS and found a greater decrease in seizure frequency in non‐lesional TLE compared to patients with structural lesions.

As epilepsy is a highly heterogeneous condition, some patients benefit from DBS, while others do not respond. Currently, however, there is limited data available regarding factors for the selection of ideal DBS candidates and brain regions to stimulate. Clinical studies are needed to investigate the association between thalamic connectivity and post‐DBS seizure reduction, and to determine whether patients with a less disrupted network benefit more from inhibition of the hippocampal‐thalamic pathway. Connectivity analysis can help in better understanding the underlying pathological network, thereby enhancing the ability to predict individual patient responses.

### 4.1 Limitations

We created a structural connectivity network based on probabilistic tractography using MRtrix3 and Freesurfer's cortical and subcortical segmentation tools with methodologic restrictions in each of them, which need to be considered. Crossing‐fibers is the best‐known problem in diffusion‐based tractography models. In this study, we used the CSD model to solve this problem. Seider et al. [47] compared different diffusion imaging models for reliability and accuracy in handling multiple crossing‐fiber regions and showed that the deconvolution model is one of the most robust methods at present. Additionally, there was an age‐related difference between healthy controls and TLE‐HS. Moreover, the sample size of all three groups was relatively small, and further studies with larger sample sizes are needed to confirm the findings of this study.

## 5 Conclusion

In summary, TLE leads to bilateral structural alterations in various thalamic nuclei, which are more pronounced in patients with HS. These results indicate that the thalamus is more intact in TLE‐MRneg. The ANT‐hippocampal pathway exhibited a progressive decrease in the number of streamlines, correlating with epilepsy duration and seizure frequency in TLE‐HS. These findings suggest that the pathway is a significant fiber tract involved in seizure propagation. Further studies are needed to investigate the relationship between structural thalamic connectivity and outcome data. Understanding the dynamics of the epileptogenic network holds promise for elucidating seizure initiation and propagation in DRE and may lead to improvements in epilepsy management by informing more targeted and effective therapeutic interventions.

## Author Contributions

**Mehmet S. Yildirim:** conceptualization, investigation, writing – original draft, methodology, formal analysis, data curation, visualization, validation, software. **Radheshyam Stepponat:** data curation, investigation, writing – review and editing, software. **Florian Ph. S. Fischmeister:** writing – review and editing, methodology, resources, validation, software. **Matthias Tomschik:** writing – review and editing. **Victor Schmidbauer:** writing – review and editing, data curation. **Farjad Khalaveh:** writing – review and editing. **Johannes Koren:** writing – review and editing, data curation, investigation. **Christoph Baumgartner:** data curation, writing – review and editing, investigation. **Ekaterina Pataraia:** writing – review and editing, data curation, investigation. **Silvia Bonelli:** data curation, writing – review and editing, investigation. **Karl Rössler:** writing – review and editing. **Gregor Kasprian:** writing – review and editing, data curation, investigation, methodology. **Christian Dorfer:** project administration, writing – review and editing, writing – original draft, conceptualization, methodology, supervision.

## Conflicts of Interest

The authors declare no conflicts of interest.

## Supporting information


**Table S1.** Main findings from the Mann–Whitney‐U test post hoc analysis.

**Table S2.** Main findings from the non‐parametric ANCOVA (Quade).

## Data Availability

Data generated or analyzed during the study are available from the corresponding author by request.
