# Supplementary material for: Decreased Structural Connectivity Between Thalamic Nuclei and Hippocampus in Temporal Lobe Epilepsy—A Diffusion Tensor Imaging‐Based Study
Source: Eur J Neurol. 2025 Jan 11;32(1):e70040. doi: 10.1111/ene.70040 (PMC11724195; doi:10.1111/ene.70040)
Supplement: Supplementary file 1 — Table S1. Main findings from the Mann–Whitney‐U test post hoc analysis. [file ENE-32-e70040-s002.docx]

**Supplementary Table 1: Main findings from the Mann-Whitney-U test post hoc analysis**

| Brain structures | Group comparison | p_FDR_-value |
| --- | --- | --- |
| Volume: Entire thalamus ipsilateral |  |  |
|  | TLE-HS vs TLE-MRneg | 0.054 |
|  | TLE-HS vs CTRL-LH | 0.002 |
|  | TLE-HS vs CTRL-RH | 0.005 |
|  | TLE-MRneg vs CTRL-LH | 0.049 |
|  | TLE-MRneg vs CTRL-RH | 0.201 |
| Volume: Entire thalamus contralateral |  |  |
|  | TLE-HS vs TLE-MRneg | 0.206 |
|  | TLE-HS vs CTRL-LH | 0.013 |
|  | TLE-HS vs CTRL-RH | 0.032 |
|  | TLE-MRneg vs CTRL-LH | 0.177 |
|  | TLE-MRneg vs CTRL-RH | 0.403 |
| Volume: ANT ipsilateral |  |  |
|  | TLE-HS vs TLE-MRneg | 0.099 |
|  | TLE-HS vs CTRL-LH | 0.013 |
|  | TLE-HS vs CTRL-RH | 0.006 |
|  | TLE-MRneg vs CTRL-LH | 0.116 |
|  | TLE-MRneg vs CTRL-RH | 0.013 |
| Volume: ANT contralateral |  |  |
|  | TLE-HS vs TLE-MRneg | 0.604 |
|  | TLE-HS vs CTRL-LH | 0.054 |
|  | TLE-HS vs CTRL-RH | 0.008 |
|  | TLE-MRneg vs CTRL-LH | 0.099 |
|  | TLE-MRneg vs CTRL-RH | 0.013 |
| Volume: MNT ipsilateral |  |  |
|  | TLE-HS vs TLE-MRneg | 0.454 |
|  | TLE-HS vs CTRL-LH | 0.006 |
|  | TLE-HS vs CTRL-RH | 0.005 |
|  | TLE-MRneg vs CTRL-LH | 0.022 |
|  | TLE-MRneg vs CTRL-RH | 0.032 |
| Volume: PNT ipsilateral |  |  |
|  | TLE-HS vs TLE-MRneg | 0.063 |
|  | TLE-HS vs CTRL-LH | < 0.001 |
|  | TLE-HS vs CTRL-RH | 0.032 |
|  | TLE-MRneg vs CTRL-LH | 0.032 |
|  | TLE-MRneg vs CTRL-RH | 0.604 |
| Volume: VNT ipsilateral |  |  |
|  | TLE-HS vs TLE-MRneg | 0.116 |
|  | TLE-HS vs CTRL-LH | 0.008 |
|  | TLE-HS vs CTRL-RH | 0.006 |
|  | TLE-MRneg vs CTRL-LH | 0.370 |
|  | TLE-MRneg vs CTRL-RH | 0.257 |
| Volume: INT ipsilateral |  |  |
|  | TLE-HS vs TLE-MRneg | 0.048 |
|  | TLE-HS vs CTRL-LH | 0.006 |
|  | TLE-HS vs CTRL-RH | 0.008 |
|  | TLE-MRneg vs CTRL-LH | 0.283 |
|  | TLE-MRneg vs CTRL-RH | 0.357 |
| Volume: HC ipsilateral |  |  |
|  | TLE-HS vs TLE-MRneg | < 0.001 |
|  | TLE-HS vs CTRL-LH | < 0.001 |
|  | TLE-HS vs CTRL-RH | < 0.001 |
|  | TLE-MRneg vs CTRL-LH | 0.444 |
|  | TLE-MRneg vs CTRL-RH | 0.420 |
| Volume: HC contralateral |  |  |
|  | TLE-HS vs TLE-MRneg | 0.275 |
|  | TLE-HS vs CTRL-LH | 0.049 |
|  | TLE-HS vs CTRL-RH | 0.038 |
|  | TLE-MRneg vs CTRL-LH | 0.214 |
|  | TLE-MRneg vs CTRL-RH | 0.144 |
| Connectivity: HC-ANT ipsilateral |  |  |
|  | TLE-HS vs TLE-MRneg | 0.008 |
|  | TLE-HS vs CTRL-LH | 0.008 |
|  | TLE-HS vs CTRL-RH | 0.006 |
|  | TLE-MRneg vs CTRL-LH | 0.444 |
|  | TLE-MRneg vs CTRL-RH | 0.604 |
| Connectivity: HC-VNT ipsilateral |  |  |
|  | TLE-HS vs TLE-MRneg | 0.076 |
|  | TLE-HS vs CTRL-LH | 0.231 |
|  | TLE-HS vs CTRL-RH | 0.635 |
|  | TLE-MRneg vs CTRL-LH | 0.005 |
|  | TLE-MRneg vs CTRL-RH | 0.080 |

ANT: Anterior thalamic nuclei

HC: Hippocampus

INT: Intralaminar thalamic nuclei

MNT: Medial thalamic nuclei

PNT: Posterior thalamic nuclei

TLE-HS: Temporal lobe epilepsy with hippocampal sclerosis

TLE-MRneg: MRI-negative temporal lobe epilepsy

VNT: Ventral thalamic nuclei
