# Supplementary material for: Decreased Structural Connectivity Between Thalamic Nuclei and Hippocampus in Temporal Lobe Epilepsy—A Diffusion Tensor Imaging‐Based Study
Source: Eur J Neurol. 2025 Jan 11;32(1):e70040. doi: 10.1111/ene.70040 (PMC11724195; doi:10.1111/ene.70040)
Supplement: Supplementary file 2 — Table S2. Main findings from the non‐parametric ANCOVA (Quade). [file ENE-32-e70040-s001.docx]

**Supplementary Table 2: Main findings from the non-parametric ANCOVA (Quade)**

| Brain structures | Group comparison | p-value |
| --- | --- | --- |
| Volume: Entire thalamus ipsilateral |  |  |
|  | TLE-HS vs TLE-MRneg | 0.155 |
|  | TLE-HS vs CTRL-LH | 0.001 |
|  | TLE-HS vs CTRL-RH | 0.004 |
|  | TLE-MRneg vs CTRL-LH | 0.044 |
|  | TLE-MRneg vs CTRL-RH | 0.21 |
| Volume: Entire thalamus contralateral |  |  |
|  | TLE-HS vs TLE-MRneg | 0.515 |
|  | TLE-HS vs CTRL-LH | 0.047 |
|  | TLE-HS vs CTRL-RH | 0.146 |
|  | TLE-MRneg vs CTRL-LH | 0.188 |
|  | TLE-MRneg vs CTRL-RH | 0.516 |
| Volume: ANT ipsilateral |  |  |
|  | TLE-HS vs TLE-MRneg | 0.452 |
|  | TLE-HS vs CTRL-LH | 0.021 |
|  | TLE-HS vs CTRL-RH | 0.002 |
|  | TLE-MRneg vs CTRL-LH | 0.181 |
|  | TLE-MRneg vs CTRL-RH | 0.016 |
| Volume: ANT contralateral |  |  |
|  | TLE-HS vs TLE-MRneg | 0.92 |
|  | TLE-HS vs CTRL-LH | 0.096 |
|  | TLE-HS vs CTRL-RH | 0.006 |
|  | TLE-MRneg vs CTRL-LH | 0.087 |
|  | TLE-MRneg vs CTRL-RH | 0.004 |
| Volume: MNT ipsilateral |  |  |
|  | TLE-HS vs TLE-MRneg | 0.968 |
|  | TLE-HS vs CTRL-LH | 0.006 |
|  | TLE-HS vs CTRL-RH | 0.006 |
|  | TLE-MRneg vs CTRL-LH | 0.007 |
|  | TLE-MRneg vs CTRL-RH | 0.01 |
| Volume: PNT ipsilateral |  |  |
|  | TLE-HS vs TLE-MRneg | 0.188 |
|  | TLE-HS vs CTRL-LH | < 0.001 |
|  | TLE-HS vs CTRL-RH | 0.098 |
|  | TLE-MRneg vs CTRL-LH | 0.011 |
|  | TLE-MRneg vs CTRL-RH | 0.893 |
| Volume: VNT ipsilateral |  |  |
|  | TLE-HS vs TLE-MRneg | 0.125 |
|  | TLE-HS vs CTRL-LH | 0.013 |
|  | TLE-HS vs CTRL-RH | 0.005 |
|  | TLE-MRneg vs CTRL-LH | 0.342 |
|  | TLE-MRneg vs CTRL-RH | 0.195 |
| Volume: INT ipsilateral |  |  |
|  | TLE-HS vs TLE-MRneg | 0.084 |
|  | TLE-HS vs CTRL-LH | 0.009 |
|  | TLE-HS vs CTRL-RH | 0.014 |
|  | TLE-MRneg vs CTRL-LH | 0.376 |
|  | TLE-MRneg vs CTRL-RH | 0.493 |
| Volume: HC ipsilateral |  |  |
|  | TLE-HS vs TLE-MRneg | < 0.001 |
|  | TLE-HS vs CTRL-LH | < 0.001 |
|  | TLE-HS vs CTRL-RH | < 0.001 |
|  | TLE-MRneg vs CTRL-LH | 0.733 |
|  | TLE-MRneg vs CTRL-RH | 0.668 |
| Volume: HC contralateral |  |  |
|  | TLE-HS vs TLE-MRneg | 0.788 |
|  | TLE-HS vs CTRL-LH | 0.151 |
|  | TLE-HS vs CTRL-RH | 0.112 |
|  | TLE-MRneg vs CTRL-LH | 0.293 |
|  | TLE-MRneg vs CTRL-RH | 0.198 |
| Connectivity: HC-ANT ipsilateral |  |  |
|  | TLE-HS vs TLE-MRneg | 0.001 |
|  | TLE-HS vs CTRL-LH | 0.014 |
|  | TLE-HS vs CTRL-RH | 0.006 |
|  | TLE-MRneg vs CTRL-LH | 0.323 |
|  | TLE-MRneg vs CTRL-RH | 0.501 |
| Connectivity: HC-VNT ipsilateral |  |  |
|  | TLE-HS vs TLE-MRneg | 0.025 |
|  | TLE-HS vs CTRL-LH | 0.14 |
|  | TLE-HS vs CTRL-RH | 0.875 |
|  | TLE-MRneg vs CTRL-LH | < 0.001 |
|  | TLE-MRneg vs CTRL-RH | 0.038 |

ANCOVA: Analysis of covariance

ANT: Anterior thalamic nuclei

HC: Hippocampus

INT: Intralaminar thalamic nuclei

MNT: Medial thalamic nuclei

PNT: Posterior thalamic nuclei

TLE-HS: Temporal lobe epilepsy with hippocampal sclerosis

TLE-MRneg: MRI-negative temporal lobe epilepsy

VNT: Ventral thalamic nuclei
